# Inhibition of *Verticillium* Wilt in Cotton through the Application of *Pseudomonas aeruginosa* ZL6 Derived from Fermentation Residue of Kitchen Waste

**DOI:** 10.4014/jmb.2401.01022

**Published:** 2024-04-05

**Authors:** Qiuhong Niu, Shengwei Lei, Guo Zhang, Guohan Wu, Zhuo Tian, Keyan Chen, Lin Zhang

**Affiliations:** 1 College of Life Science and Agricultural Engineering, Nanyang Normal University, 1638 Wolong Road, Nanyang, Henan 473061, P.R.China; 2 College of Agriculture and Engineering, Nanyang Vocational College of Agriculture, Nanyang, Henan 473000, P.R. China

**Keywords:** Biocontrol, cotton, kitchen waste, *Pseudomonas*, *Verticillium dahliae*, *Verticillium* wilt

## Abstract

To isolate and analyze bacteria with *Verticillium* wilt-resistant properties from the fermentation residue of kitchen wastes, as well as explore their potential for new applications of the residue. A total of six bacterial strains exhibiting *Verticillium* wilt-resistant capabilities were isolated from the biogas residue of kitchen waste fermentation. Using a polyphasic approach, strain ZL6, which displayed the highest antagonistic activity against cotton *Verticillium* wilt, was identified as belonging to the *Pseudomonas aeruginosa*. Bioassay results demonstrated that this strain possessed robust antagonistic abilities, effectively inhibiting *V. dahliae* spore germination and mycelial growth. Furthermore, *P. aeruginosa* ZL6 exhibited high temperature resistance (42°C), nitrogen fixation, and phosphorus removal activities. Pot experiments revealed that *P. aeruginosa* ZL6 fermentation broth treatment achieved a 47.72% biological control effect compared to the control group. Through activity tracking and protein mass spectrometry identification, a neutral metalloproteinase (*Nml*) was hypothesized as the main virulence factor. The mutant strain ZL6Δ*Nml* exhibited a significant reduction in its ability to inhibit cotton *Verticillium* wilt compared to the strain *P. aeruginosa* ZL6. While the inhibitory activities could be partially restored by a complementation of *nml* gene in the mutant strain ZL6CMΔ*Nml*. This research provides a theoretical foundation for the future development and application of biogas residue as biocontrol agents against *Verticillium* wilt and as biological preservatives for agricultural products. Additionally, this study presents a novel approach for mitigating the substantial amount of biogas residue generated from kitchen waste fermentation.

## Introduction

In 2020, the yield of kitchen waste in China reached approximately 1.4 billion tons [1], and as the population and economy continue to grow, the amount of kitchen waste continues to increase [2]. Kitchen waste therefore represents a significant source of renewable energy [3], and finding renewable technologies to address the growing amount of kitchen waste is crucial. The pH of kitchen digestate ranges from 7.3 to 8.9, and this pH change occurs due to the degradation of volatile fatty acids and the reduction of multivalent ions during the anaerobic digestion process. Kitchen digestate is abundant in organic matter, with a content ranging from 61.7% to 73.6%(on a dry basis). Additionally, it is also rich in nutrients such as nitrogen, phosphorus, and potassium [4]. The direct discharge of a large amount of biogas residue can easily cause environmental pollution, and the costs of standard treatment are high. Thus, a reasonable recycling and treatment method is urgently needed.

Anaerobic digestion is the most important method of processing food waste. Large amounts of biogas slurry and biogas residues pose considerable challenges to society. We reduce this hazard through aerobic composting and produce organic fertilizer. However, there is still insufficient research on the combination of biogas residue and biocontrol bacteria to produce bioorganic fertilizers that can prevent and control crop diseases. In fact, it has been reported that biogas residue improves microbial diversity and disease suppression in indigenous soil microbial biomass. Moreover, the application of biogas residue leads to a higher level of indigenous soil microbial biomass and a lower abundance of potential fungal pathogens in both bulk and rhizosphere soil compared with chemical fertilizer [5]. The combination of functional microorganisms and agricultural solid waste in producing bioorganic fertilizers for controlling soil-borne diseases and achieving sustainable development is of great practical significance [6, 7]. Thus, the use of biogas residue to produce bioorganic fertilizer not only offers a solution for disposing of a large amount of anaerobic digestion residue, but it also generates additional income, making it one of the most promising choices for agricultural production.

*Verticillium* wilt is one of the most critical diseases affecting cotton due to its extensive transmission routes, severe damage, and complex infection mechanism [8], it is widely distributed in cotton-producing countries [9]. Cotton *Verticillium* wilt is primarily caused by the soil fungus *V. dahliae* [10] and results in the wilting, fading, and shedding of cotton leaves [11, 12]. Moreover, it slows down or even halts the growth and development of cotton, leading to a decline in cotton quality and yield [13]. The average yield reduction caused by *Verticillium* wilt in cotton is approximately 10%-35% [14]. Therefore, the effective control of *Verticillium* wilt is crucial in cotton disease resistance research.

Controlling *Verticillium* wilt disease is challenging due to the long persistence of resting structures in the field and the broad host ranges of some species [10]. Disease management strategies include breeding resistant cultivars, altering agricultural planting patterns, applying chemical fungicides, and implementing crop rotation [15]. Unfortunately, there are several concerns about these methods and they have some limitations; they have also proven to be less effective and environmentally friendly than expected [16]. Currently, the use of biological control agents is a promising and environmentally friendly strategy for controlling *Verticillium* wilt in cotton [17]. An increasing number of bacteria are demonstrating promising abilities to control *Verticillium* wilt in laboratory and greenhouse experiments due to their pervasiveness, experimental tractability, and impact on ecosystems, economies, agriculture, and human health.

The aim of this study was to develop a microbial agent that can help cotton resist *Verticillium* wilt. In order to obtain biocontrol strains, strains capable of inhibiting the pathogen causing cotton *Verticillium* wilt were screened and identified from digestate residues. After identifying the bacterial species, the biocontrol effects were verified and the molecular mechanism for resistance against cotton *Verticillium* wilt was explored to develop microbial agents. The results of this study provide new insights into the mode of action of potential biogas residue biocontrol agents, contributing to the realization of sustainable development and the promotion of eco-friendly methods for controlling *Verticillium* wilt.

## Materials and Methods

### Microbial Strains, Plasmids, and Materials

The fungus *V. dahliae* V991 was provided by the State Key Laboratory of Cotton Biology at Henan University (Kaifeng, China). The antagonistic strain *P. aeruginosa* ZL6, which was isolated from the fermentation residue of kitchen waste, was deposited in the China Center for Type Culture Collection (CCTCC M20221084). The plasmid pJQ200SK, which was used for gene knockout, was obtained from the MiaoLing Center of Biological Resource Collection.

The fungi were cultivated on potato dextrose agar (PDA) plates (20.0% potato, 2.0% glucose, and 1.5% agar), while the bacterial strains were incubated on Luria-Bertani (LB) culture medium containing 1.0% tryptone, 0.5%yeast extract, and 0.5% NaCl, supplemented with selective antibiotics. All of the chemical reagents used in this study were purchased from Sigma (USA).

A ClonExpress II One step Cloning Kit (Vazyme, China) was used for all plasmid constructions. Plasmid Extraction and Purification Kits were purchased from OMEGA (USA), while PrimeSTAR Max DNA Polymerase and restriction endonucleases and high-fidelity restriction endonucleases were acquired from Takara (Japan).

### Screening and Identification of Bacterial Strains Antagonistic against *V. dahliae*

Screening and identification of biocontrol strains against *V. dahliae* were conducted following the methods described in a previous study [18]. Biogas residue samples were collected from food waste and naturally dried for one month. Ten grams of the samples were taken and added to 100 ml of liquid LB culture medium for 2 h. After dilution, gradient dilutions were spread onto LB plates and incubated for 12 h at 37°C. Single colonies with different shapes, sizes, and colors were purified, numbered, and stored at 4°C. Antifungal activities were evaluated using the confrontation culture method, in which the center of a PDA medium plate was inoculated with *V. dahliae* and cultured at 25°C for 3 d. The ZL6 bacteria was cultured in 5 mL of LB liquid medium at 37°C and 220 rpm for 12 h. A 100 μl aliquot was spread onto an LB agar plate and incubated at 37°C for 12 h. Subsequently, a bacterial block with a diameter of 1 cm was extracted using a punch and placed on the PDA plate inoculated with *V. dahliae*. A blank LB block was symmetrically placed on the other side of the PDA plate as a negative control. The growth of fungal mycelia was observed over 5 d at a temperature of 25°C, during which the colony diameter was measured every day. The antifungal rate was calculated using the previous publication [19].

### Whole-Genome Sequencing of ZL6

Whole-genome sequencing and analysis of strain ZL6 were conducted following methods previously described in the literature [20]. The whole-genome sequencing was performed by Shanghai Majorbio Bio-Pharm Technology Co., Ltd using Illumina Hiseq and PacBio. Six major databases (NR, Swiss-Prot, Pfam, EggNOG, GO and KEGG) were used for gene function annotation. Secondary metabolite biosynthetic gene clusters were predicted using the antiSMASH bacterial version. Protein-coding genes of the bacterium were predicted using GeneMarkS software (http://topaz.gatech.edu/) while leveraging information from Swiss-Prot (http://web.expasy.org/docs/swiss-prot_guideline.html). Genomic islands (GI) were identified using Island PathDIOMB, and prophages were predicted using phiSpy. In addition, non-coding RNAs (ncRNAs), including sRNA, rRNA, tRNA, snRNA, and miRNA, were predicted through the use of BLAST searches against the Kyoto Encyclopedia of Genes and Genomes (KEGG, http://www.kegg.jp/) rRNA database, as well as with rRNAmmer, tRNAscan, and Rfam.

### Purification of the Inhibition Factor and Mass Spectrum Identification

The characteristics of the antifungal metabolites produced by strain ZL6 were tested following the methods described in a previous publication [21]. The 500 ml cultures containing 3.6 × 10^9^ CFU/ml of strain ZL6 were centrifuged at 8000 rpm for 15 min, and the supernatant was then collected and subjected to gradient ammonium sulfate saturation (0-20%, 20-30%, 30-40%, 40-50%, 50-60%, 60-70%, 70-80%) by slow continuous stirring at 4°C. The treated dialysis bag was used for 16 h of dialysis to remove excess salt, and the inhibitory activity of each fraction against *V. dahliae* was tested. Aliquots of 200 ml from the tested fractions and untreated control were separately placed into holes at equal distances (2 cm) from the center of the *V. dahliae* plate. The suspension was then incubated at 25°C for 5 d, and the diameter of the inhibition zone was measured. The experiment was repeated three times.

Sodium dodecyl sulfate polyacrylamide gel electrophoresis (SDS-PAGE) was performed in order to examine the purified protein. The protein sample treatment process for mass spectrometry identification was carried out according to the literature [21]. The protein gel slice was excised using a surgical knife blade and transferred to V-bottom 96-well microplates loaded with 100 μl of 50% acetonitrile (ACN)/25 mM ammonium bicarbonate solution per well. After 1 h of destaining, the gel plugs were dehydrated with 100 μl of 100% ACN for 20 min, followed by complete drying in a SpeedVac concentrator (Thermo Savant, USA) for 30 min. The dried gel particles were rehydrated at 4°C for 45 min with 2 μl/well of trypsin (Promega, USA) in 25 mM ammonium bicarbonate before being incubated at 37°C for 12 h. The peptide mixtures were extracted with 20 μl of a solution containing 50% ACN/0.5% trifluoroacetic acid (TFA) per well at 37°C for 1 h after digestion with trypsin. Finally, the extracts were dried under N2 gas. The peptides were eluted with 0.8 μl of a matrix solution [α-cyano-4-hydroxy-cinnamic acid (CHCA), Sigma, USA] in 0.1% TFA and 50% ACN before being spotted onto the target plate. The samples were air-dried and analyzed using a 5800 matrix-assisted laser desorption/ionization-time of flight (MALDI-TOF/TOF) Proteomics Analyzer (Applied Biosystems, USA). The MALDI-TOF/TOF MS and MS/MS analysis, as well as database searches, were conducted with the assistance of GeneCore Company (China).

### Cloning, Expression, Knockout and Complementation of nml

All primers used in this study are described in Table 1. The primers (CNm-F and CNm-R) were designed based on the genome sequencing and NCBI sequence alignment. The PCR amplification was performed under the following conditions: initial denaturation at 98°C for 4 min, followed by 30 amplification cycles at 98°C for 10 s, 62°C for 10 s, and 72°C for 1 min, and a final extension at 72°C for 10 min. The PCR products were cloned into the pUC19 vector using the One-Step Clone Kit and were then subsequently sequenced. The deduced amino acid sequences were aligned with other previously reported neutral metalloproteinases, and MEGA5.0 software was used to construct a phylogenetic tree of Nml.

To construct gene expression vector (pET-Nml), PCR amplification was conducted using primers (TNm-F and TNm-R) under the same conditions. The amplified fragment was ligated into the pET-32a expression vector, which had been double-digested with HindIII and BamHI restriction enzymes. After verifying the reading frame through sequencing, the plasmid pET-Nml was transferred into *E. coli* BL21 for heterologous expression. A single colony containing the plasmid pET-Nml was obtained and inoculated into 5 ml of LB liquid medium, which was shaken at 37°C for 12-16 h. The bacterial culture was then mixed with 200 ml of LB medium at a ratio of 1:100. The transformed cells were cultured until they reached an optical density (OD_600_) of 0.6-0.8. After optimizing the induction conditions, including time, temperature, and IPTG concentration, the recombinant proteins were purified using the One-Step His-Tagged Protein Miniprep Pack (Biomart, China) according to the provided protocol.

To construct the gene *nml* disruption mutant strain, the suicide plasmid pJQ200SK was utilized following a previously published protocol [22]. A fragment of approximately 525 bp near the 5’ end was amplified via PCR using the primers (KNm-F and KNm-R). The amplified fragment was fused with the SacI- and BamHI-digested pJQ200SK plasmid using a one-step cloning kit, resulting in the generation of pJQ200SK-Nml. The plasmid pJQ200SK-Nml was then introduced into strain ZL6 through triparental mating with *E. coli* HB101 (pRK600) serving as the helper strain. The candidate mutants were screened on 1/5 LB agar supplemented with 100 μg/ml streptomycin and 15 μg/ml gentamicin. The gene *nml* disruption mutant strain (ZL6Δ*Nml*) was confirmed via PCR and sequencing.

For complementation studies, the complete 1494-bp DNA fragment of *nml* gene was amplified from the genome of ZL6. Subsequently, it was inserted into the KpnI-EcoRV site of pBBR1MCS-2 plasmid, generating the complementation plasmid designated as pBBR*Nml*ZL6. The knockout mutant ZL6Δ*Nml* was then transformed with the pBBR*Nml*ZL6 plasmid via triparental conjugative transfer. Positive clones were selected on LB agar plates containing kanamycin, verified through PCR, and confirmed as ZL6CMΔ*Nml*.

### Antifungal Activity Test in Potting Experiments

The wild-type strain ZL6, the mutant strains ZL6Δ*Nml* and the mutant strain ZL6CMΔ*Nml* were cultured overnight in LB liquid medium at 37°C and 220 rpm. The bacteria were collected through centrifugation at 8000 rpm for 5 min and adjusted to an optical density of OD_600nm_ = 1.0 using sterile water for use in the potting experiments.

The potting tests were conducted following a previously published protocol [18]. A spore suspension of the fungus *V. dahliae* V991 was prepared using Czapek Dox liquid medium, and the spore quantity was determined with a hemocytometer. The spores were then mixed with the soil mixture (loess/black soil/vermiculite at a ratio of 1:1:1) to obtain spore soil with a spore concentration of 1 × 10^6^ spores/g. The spore soil mixture was placed into a square bowl measuring 7 cm × 7 cm × 7 cm. Four groups were set up: the first group consisted of spore soil mixed with 100 ml of sterile water; the second group consisted of spore soil mixed with 10 ml of ZL6 suspension (OD_600nm_ = 1.0) and 90 mL of sterile water; and the third group consisted of spore soil mixed with 10 ml of ZL6Δ*Nml* suspension (OD_600nm_ = 1.0) and 90 ml of sterile water; and the four group consisted of spore soil mixed with 10 mL of ZL6CMΔ*Nml* suspension (OD_600nm_ = 1.0) and 90 ml of sterile water.

Cotton seeds were planted in the four groups of soil and cultured at 25°C in a light incubator with a light/dark cycle of 16/8 h. The disease index (DI) levels were determined and recorded every 5 d after 30 d of incubation and were based on the following criteria: healthy plants = 0, one cotyledon or part of a cotyledon turning yellow or necrotic = 1, two cotyledons turning yellow or necrotic = 2, one true leaf turning yellow or necrotic = 3, and two or more true leaves turning yellow or necrotic = 4. The final DI value was calculated using the following formula: DI = (∑ (number of stages × number of plants) / (highest level value × total number of plants)) × 100%. Each pot trial was repeated three times in triplicate.

### DNA Preparation and Metagenomic Analysis of Microbiota

The tested biogas residue group with the bacterium ZL6 and the control group (CK) without inoculated bacteria were fermented for 60 d respectively, and the total DNA of the two groups were extracted using a PowerSoil DNA Isolation Kit (MoBio, USA) following the manufacturer’s protocols. The concentration and purity of the extracted DNA were determined using a TBS-380 and NanoDrop2000, respectively. The quality of the extracted DNA was evaluated on 1% agarose gel. The DNA was fragmented to an average size of approximately 300 bp using a Covaris M220 (Gene Company Limited, China) for paired-end library construction.

The paired-end library was constructed using a NEXTFLEX Rapid DNA-Seq kit (Bioo Scientific, Austin, TX, USA). Adapters containing the full complement of sequencing primer hybridization sites were ligated to the blunt ends of the DNA fragments. Paired-end sequencing was performed on an Illumina NovaSeq (Illumina Inc., USA) at Majorbio Bio-Pharm Technology Co., Ltd. (China) using NovaSeq Reagent Kits according to the manufacturer’s instructions (www.illumina.com). Adapter sequences were removed from the 3' and 5' ends of the paired-end Illumina reads using SeqPrep (https://github.com/jstjohn/SeqPrep), and low-quality reads (length < 50 bp, quality value < 20, or containing N bases) were filtered out using Sickle (https://github.com/najoshi/sickle). The metagenomic data were assembled using MEGAHIT (https://github.com/voutcn/megahit) [23], which utilizes succinct de Bruijn graphs. Contigs with a length of ≥300 bp were selected as final assemblies and used for further gene prediction and annotation [24, 25].

## Results

### Isolation and Identification of Strain ZL6 with Antagonistic Activity against *V. dahliae*

**Fig. 1:**
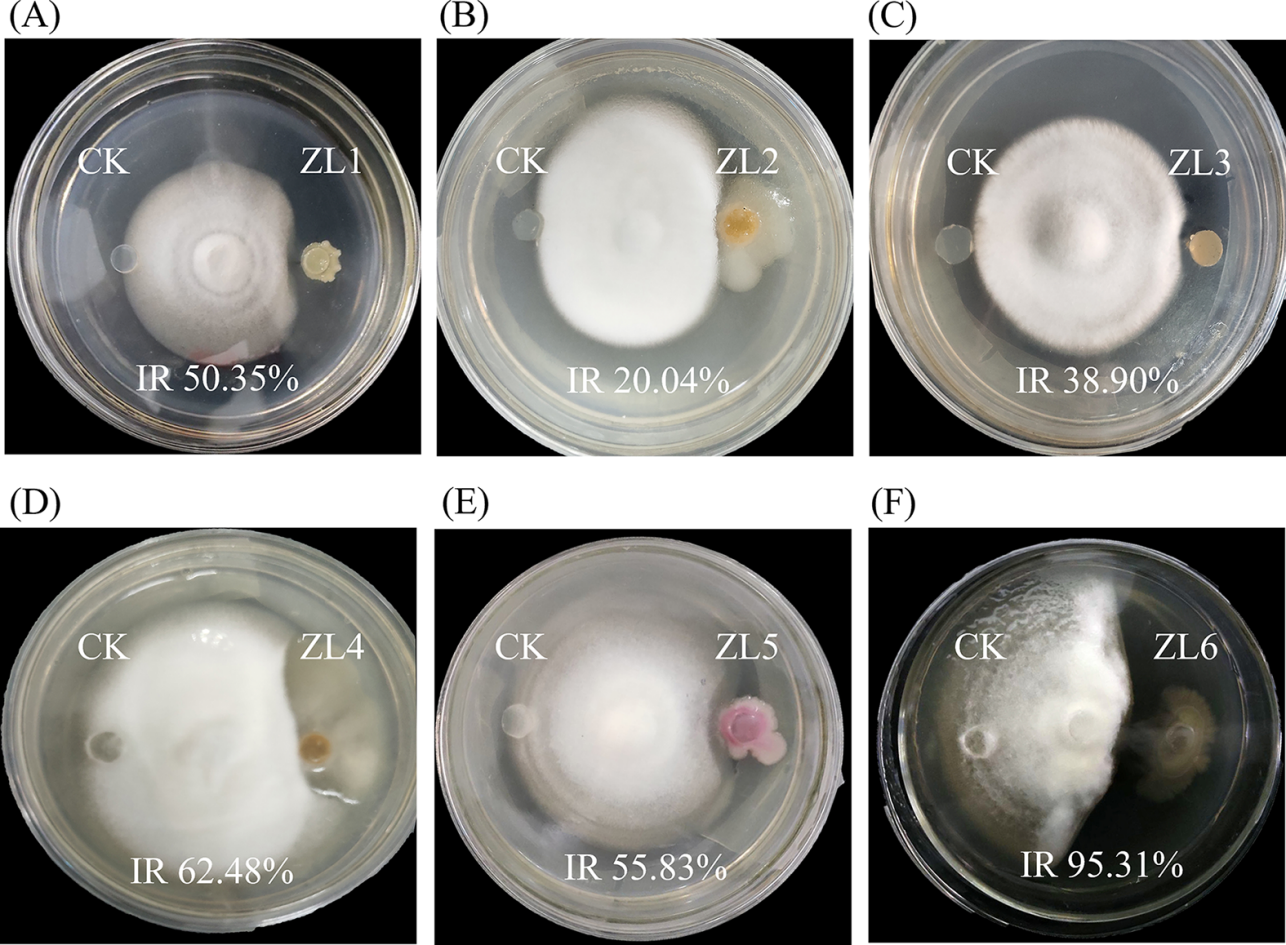
Anti-fungal activity results of the six bacterial strains tested by plate inhibition experiments.

**Fig. 2:**
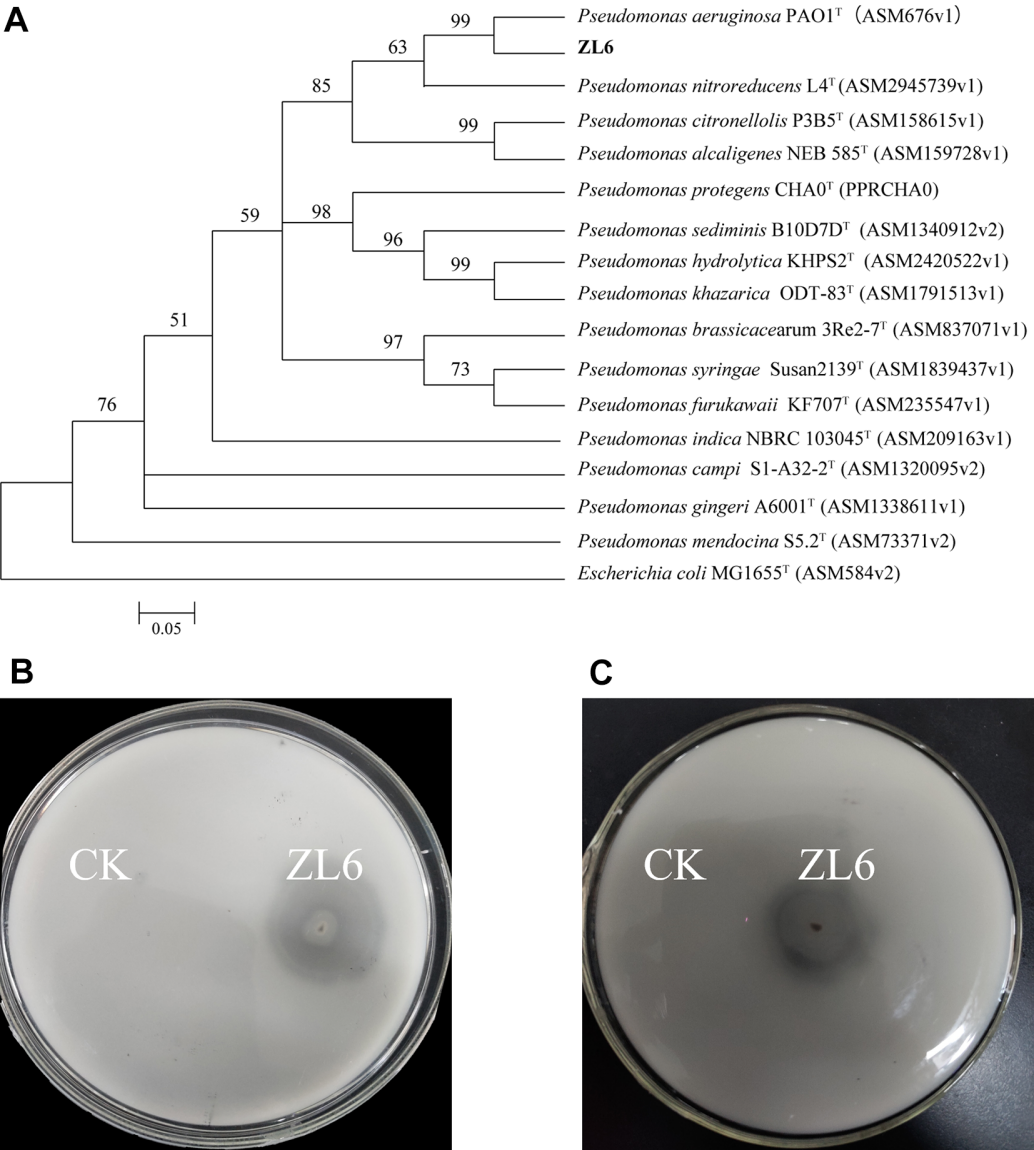
(A) Neighbor-joining tree based on 16S rRNA gene sequences showing the relationship of the strain ZL6 with validly described species in the genus *Pseudomonas* and other related taxa. (B) Test results of the nitrogen fixation activity of the strain ZL6. (C) Test results of the phosphorus solubilization activity of the strain ZL6. Notes: On the left side, CK is *Escherichia coli*, and on the right side, ZL6 is a single colony.

A total of 73 bacteria were isolated from the fermentation residue of the kitchen waste, and among them, 44 bacteria were able to grow at a temperature of 40°C. Six bacterial strains, ZL1-ZL6, exhibited relatively strong fungistatic activities against the plant pathogenic fungus *V. dahliae* and were capable of growing at 40°C (Fig. S1). Plate confrontation experiments revealed the inhibition rates of ZL1-ZL6 against *V. dahliae* to be 50.35%, 20.04%, 38.90%, 62.48%, 55.83%, and 95.31%, respectively. Among these six strains, ZL6 exhibited the most significant inhibitory activity against the *Verticillium* wilt pathogenic fungus *V. dahliae* (Fig. 1). The strain was deposited in a publicly accessible culture collection under the accession number CCTCC No. M20221084. Identification of the six strains was based on their morphological properties and analysis of their 16S rRNA sequences. The results showed that these six strains belonged to *Pseudomonas* species, *Exiguobacterium* species, *Stenotrophomonas* species, *Stenotrophomonas* species, and *Microbacterium* species, respectively (Table S1). The nearly full-length sequence of the 16S rRNA gene (1,543 bp) of strain ZL6 was obtained. A BLAST search in GenBank revealed that the 16S rRNA gene of strain ZL6 exhibited 100% identity to the 16S rRNA genes of members of the genus *Pseudomonas* (Fig. 2A). Comparison of nucleotide sequences results showed the most closely related strain was *P. aeruginosa* PAO1^T^, which had 100% identity. The strains ZL2, ZL5, and ZL6 exhibited positive nitrogen fixation activities. The strains ZL5 and ZL6 showed positive phosphorus solubilization activities, and the strains ZL3 and ZL5 had potassium solubilization activities (Table S1). The nitrogen fixation activity test showed that strain ZL6 exhibited a clear transparent zone, with the ratio of the diameter of the nitrogen fixation zone to that of the bacterial colony being approximately 0.8 (Fig. 2B). The phosphorus solubilization activity test results indicated that strain ZL6 produced a clear transparent zone, indicating strong phosphorus solubilization ability. The ratio of the diameter of the phosphorus solubilization zone to the diameter of the bacterial colony was 0.4 (Fig. 2C). These results highlight the diversity of microorganisms in biogas residue, which can serve as a potential functional bio-organic fertilizer due to their richness in heat-resistant and plant disease-resistant biocontrol strains.

### Whole-Genome Analysis of the ZL6 Strain

Whole-genome analysis of the ZL6 strain yielded a total of 148,159 clean reads, with an average read length of 7,649.22 bp. The assembly resulted in a complete circular DNA genome with a size of 6,306,714 bp and a GC% of 66.51%. Gene annotation identified a total of 5,716 genes (89.85% of the genome) located within the genome sequence. NcRNAs, including 64 tRNAs, four 5S rRNAs, four 16S rRNAs, four 23S rRNAs, and 132 sRNAs (total length: 13,523 bp), were predicted. Tandem repeat sequences (51 sequences, 0.4% of the genome) and interspersed repeat sequences (22 sequences, 0.05% of the genome) were also identified, and seven genomic islands (GIs), one prophage, and seven CRISPR-Cas sequences were detected. Functional annotation analysis using various databases [Non-redundant (Nr), SwissProt, Pfam, Clusters of Orthologous Genes (COG), GO, Kyoto Encyclopedia of Genes and Genomes (KEGG)] revealed the functions of the identified genes. Protein functional annotation analysis based on the COG database indicated that the identified genes were mainly involved in functions such as amino acid transport and metabolism, signal transduction mechanisms, lipid transport and metabolism, coenzyme transport and metabolism, carbohydrate transport and metabolism, secondary metabolite biosynthesis, transport and catabolism, defense mechanisms, nucleotide transport and metabolism, cell motility, and extracellular structures.

The GO functional analysis showed that 75.86% of the genes were assigned to the Molecular Function (MF), Cellular Component (CC), and Biological Process (BP) ontologies, while the KEGG annotation revealed the involvement of the identified genes in cellular processes, environmental information processing, genetic information processing, human disease, metabolism, and organismal system-related pathways. Pathogen analysis identified 930 virulence factor (VF)-related genes, including adherence factors, iron uptake systems, secretion systems, antiphagocytic factors, regulatory factors, toxins, serum resistance factors, stress proteins, invasion factors, magnesium uptake systems, phase variation factors, and complement proteases based on the Virulence Factor Database (VFDB).

A total of 368 drug-resistant genes were identified, including resistance to tetracycline antibiotics, fluoroquinolone antibiotics, macrolide antibiotics, penams, cephalosporins, phenicol antibiotics, and aminoglycoside antibiotics. The PHI analysis clustered 1,497 genes into categories such as reduced virulence, unaffected pathogenicity, increased virulence (hypervirulence), loss of pathogenicity, effectors (plant avirulence determinant), lethal factors, and resistance/sensitivity to chemicals. Based on the ResFinder database, five drug resistance genes, including aminoglycoside, fosfomycin, phenicol, and Beta-lactam resistance genes, were predicted. The genome analysis identified 90 genes involved in secretory systems, including Type I, II, III, VI secretion systems, Sec-SRP, and tat systems. A total of 1,278 transfer proteins and 1,385 transmembrane proteins were detected. The ZL6 genome contained 124 two-component regulatory systems, including sensor and hybrid genes. An overview of the ZL6 genome is provided in Fig. S2, and the genome sequence data of strain ZL6 have been deposited in the GenBank database with the Bio Project ID PRJNA962678.

A phylogenomic tree based on the core genomes of strains ZL6, *P. aeruginosa* PAO1, *P. thermotolerant* J53^T^ and strains of related species of the genus *Pseudomonas* as identified by KSNP 3.0 (Fig. S3). Combined with the 16S rRNA identification, ZL6 could be identified as *P. aeruginosa*.

### Purification and Identification of the Inhibition Factor

The plate inhibition assay confirmed that *P. aeruginosa* ZL6 primarily uses secreted proteases to inhibit the growth of the pathogenic fungus *V. dahliae*. This was evident from the observation that the culture supernatant of *P. aeruginosa* ZL6 exhibited inhibitory activity against *V. dahliae*, while boiled and proteinase K-treated culture supernatant proteins showed no inhibitory activity. To purify proteins with inhibitory activity from the culture supernatant of *P. aeruginosa* ZL6, a gradient ammonium sulfate precipitation method was employed.

**Fig. 3:**
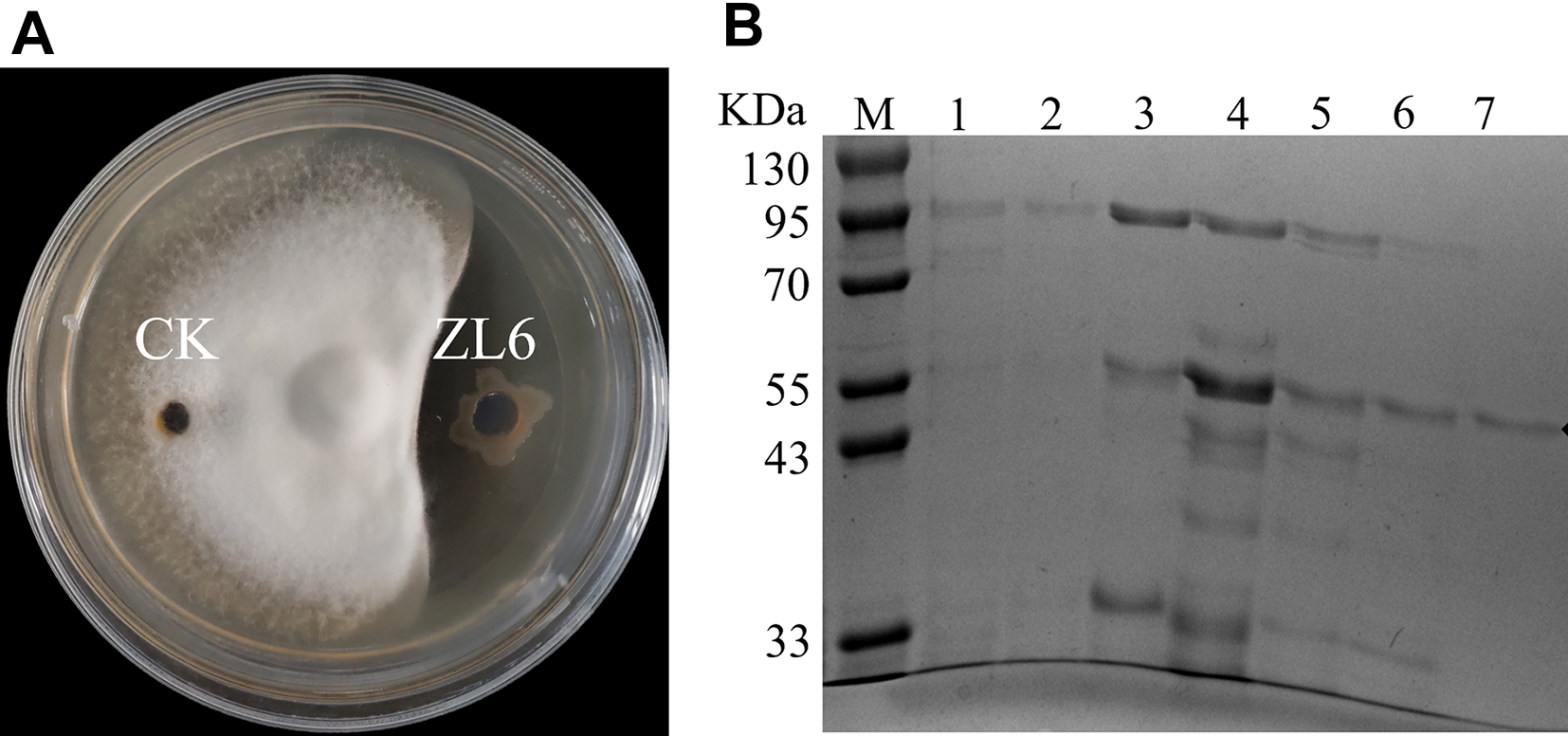
Results of the plate inhibition assays and purification process of active protein. (**A**) The effect of the purified protein on fungal growth inhibition. (**B**) SDS-PAGE of the purification process of active protein. Lanes: M, protein marker; 1. Fractions of 0-20% ammonium sulfate fractional precipitation; 2. Fractions of 20-30% ammonium sulfate fractional precipitation; 3. Fractions of 30-40% ammonium sulfate fractional precipitation; 4. Fractions of 40-50% ammonium sulfate fractional precipitation; 5. Fractions of 50-60% ammonium sulfate fractional precipitation; 6. Fractions of 60-70% ammonium sulfate fractional precipitation; 7. Fractions of 70-80% ammonium sulfate fractional precipitation purified protein.

A bioactivity tracking test was conducted for each fraction obtained during the purification process. The results revealed that all the components, except those in the 0-20% and 20-30% gradient fractions, exhibited antifungal rate s against *V. dahliae* in vitro. The purified protein fraction from the 40-50% gradient also demonstrated noticeable inhibition of *V. dahliae* growth, and the level of inhibition activity was positively correlated with the protein amount (Fig. 3A). In addition, SDS-PAGE analysis of the corresponding fraction showed a single protein band with a molecular mass of approximately 63 kDa (Fig. 3B, line 4).

To identify the protein, the purified band from *P. aeruginosa* ZL6 was excised from the SDS-PAGE gel and subjected to in-gel trypsin digestion, followed by MALDI-TOF/TOF identification. The amino acid sequence alignment analysis and annotations from protein databases, including the UniProt knowledgebase (Swiss-Prot/TrEMBL) and Gene Ontology (GO) database, suggested that the protein could be identified as a neutral metalloproteinase known as Nml.

### Cloning, Heterologous Expression, and Function Validation of the Gene nml

The deduced protein sequence is comprised of 498 amino acids. By performing a BLAST search against the NCBI database, it was found that the amino acid sequence of Nml showed the highest identity (99%) with the M4 family elastase LasB from *P. aeruginosa*. A phylogenetic tree based on the amino acid sequence was then constructed, confirming that Nml belongs to the M4 family elastase, with LasB from *P. aeruginosa* being its closest relative (HBP0264571.1). A peptidase M4 family protein (MCO3322300.1) from *P. aeruginosa* was selected as an outgroup for the phylogenetic analysis (Fig. S4).

The plasmid pET32a-Nml was successfully constructed for heterologous expression and transferred into *E. coli* BL21 competent cells. After optimizing the induction conditions, the recombinant protein rm-Nml, with an approximate molecular weight of 65 kDa, was purified using an Ni-NTA column from Qiagen. Protease and antifungal activity assays demonstrated that rm-Nml possesses protease activity and can inhibit the growth of *V. dahliae*. The antifungal rate on fungi was found to be proportional to the concentration of rm-Nml (Table 2).

**Fig. 4:**
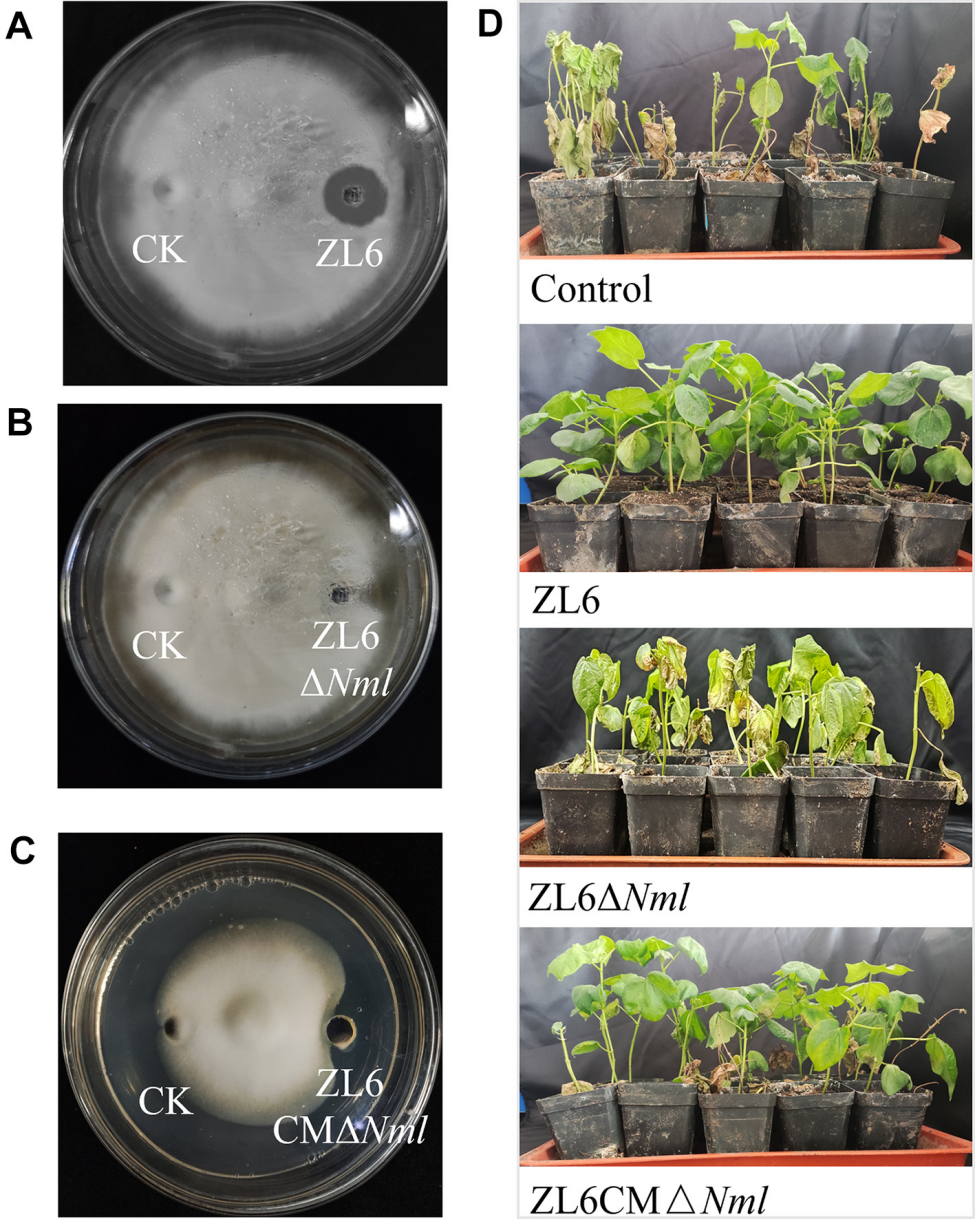
Results of wild-type and mutant strains on the inhibition of the fungus *V. dahliae*. (**A**) Antifungal activity test of wild-type *P. aeruginosa* ZL6 for 48 h on plates; (**B**) Antifungal activity test of the mutant strain ZL6Δ*Nml* for 48 h on plates. (**C**) Antifungal activity test of the mutant strain ZL6CMΔ*Nml* for 48 h on plates. (**D**) Results of potting test observations: control cotton group without inoculation of biocontrol bacteria; cotton group inoculated with the wild type *P. aeruginosa* ZL6 strain; and cotton group inoculated with the mutant strains ZL6Δ*Nml* and ZL6CMΔ*Nml*.

In inhibition activity assays, extracts from the wild-type strain *P. aeruginosa* ZL6 and the knockout mutant strain ZL6Δ*Nml* showed significantly different abilities to inhibit *V. dahliae* both on plates and in pots. In the in vitro antifungal plate activity test, clear inhibition zones were observed around the fermentation broth of the wild-type strain *P. aeruginosa* ZL6 when spread on PDA plates inoculated with *V. dahliae* (Fig. 4A). In contrast, the mutant strain ZL6Δ*Nml* and the negative control did not form clear inhibition zones on the plates (Fig. 4B). On the contrary, the complementation partially restored inhibitory activity, even though it remained comparatively lower than that observed in the wild-type strain *P. aeruginosa* ZL6 (Fig. 4C). Pot experiments confirmed that cotton plants irrigated with the wild-type strain *P. aeruginosa* ZL6 fermentation solution exhibited strong growth and increased resistance to the disease compared with the control plants infected only with V. dahlia. Nevertheless, the disease symptoms and degree of chlorosis in the plants notably ameliorated following treatment with the complementary strain ZL6CMΔ*Nml* (Fig. 4D). The infection rates of the cotton group irrigated with the wild-type *P. aeruginosa* ZL6 strain decreased by 18% to 22% compared to the control group, which showed a 100% incidence of disease. In addition, the cotton plants under the *P. aeruginosa* ZL6 treatment exhibited faster growth compared with the cotton in the control group infected with *V. dahliae*. However, the cotton group under the mutant strain ZL6Δ*Nml* showed the same disease symptoms, and the average control effect of the ZL6Δ*Nml* strain reached only 20.35%, which is significantly lower than the control rate of the wild-type strain (94.33%). In contrast, the cotton group under the complementation mutant strain ZL6CMΔ*Nml* did not show significant symptoms of wilt disease, which was very similar to the cotton group treated with the wild strain *P. aeruginosa* ZL6.

### Microbiota Community Structure Analysis during the Biogas Residue Fermentation Process Added into *P. aeruginosa* ZL6

**Fig. 5:**
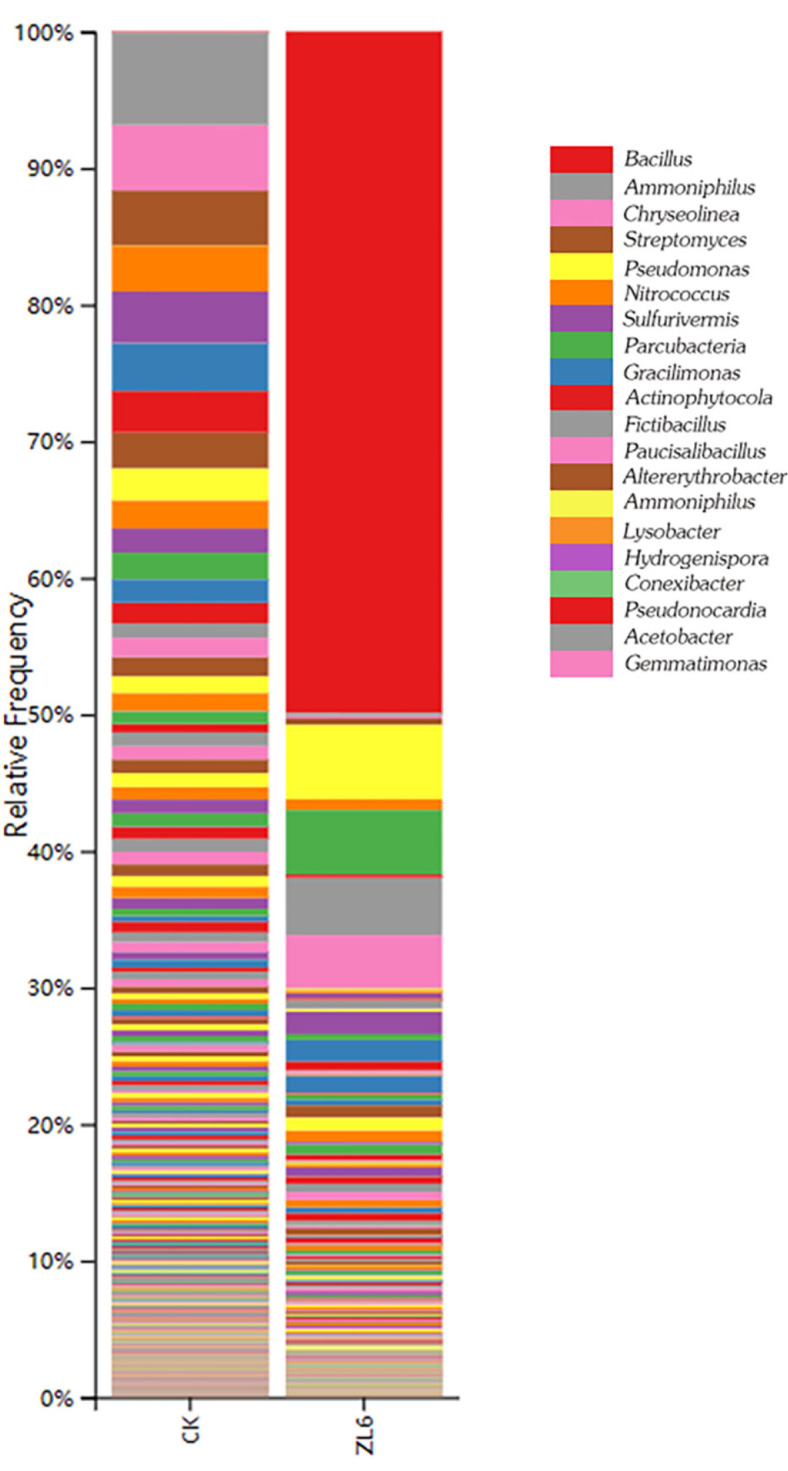
Results of the microbiota structure in the residues before and after being inoculated by *P. aeruginosa* ZL6 at the genus level by column diagram according to the metagenomic sequence analysis. Because there are many species at the genus level, only the top 20 species were selected for analysis.

During the fermentation process of biogas residue, microbiota community structure analysis was performed on two groups: the control group (CK) and the group inoculated with the strain *P. aeruginosa* ZL6. A total of 204,117 and 331,009 sequences were obtained from the CK and ZL6 groups, respectively, resulting in 19 and 25 operational taxonomic units (OTUs). The taxonomic analysis revealed significant differences in the diversity and distribution of microbiota between the control biogas residue and those inoculated with the *P. aeruginosa* ZL6 strain for 60 d. Compared with the original residue, the bacterial diversity was greatly reduced after inoculating the *P. aeruginosa* ZL6 strain and undergoing fermentation (Fig. 5). In the control group (CK), the microbiota community structure was predominantly composed of genera such as *Bacillus*, *Ammoniphilus*, *Chryseolinea*, *Pseudomonas*, *Sulfurivermis*, *Streptomyces*, *Gracilimonas*, *Paucisalibacillus*, *Fictibacillus* and *Altererythrobacter*. The ratio of undefined species to defined species is 3:7. Compared with the control group, the content of *Pseudomonas* displayed in yellow in Fig. 5 increased significantly after adding the bio-organic fertilizer, which indicated successfully colonization of the bacteria into plant roots and enabled them to reproduce in large quantities. After inoculating the *P. aeruginosa* ZL6 strain and during the fermentation process, the microbiota community underwent reconstruction, where the bacterial diversity in the ZL6 group was drastically reduced. However, the abundance of *Pseudomonas* and *Bacillus* both increased dramatically. The abundances of *Streptomyces*, *Fictibacillus* and *Paucisalibacillus* showed slight increases, while the abundances of *Ammoniphilus*, *Parcubacteria* and *Sulfurivermis* all decreased to varying extents. These results suggest that the strain *P. aeruginosa* ZL6 successfully proliferated in the fermented biogas residue and may have directly participated in, or induced other bacteria in the community to participate in, the colonization process.

## Discussion

There are several potential benefits of using biogas residue as a functional manure to inhibit pathogens and improve soil fertility. Biogas residue, which is obtained from the anaerobic digestion of organic waste, is increasingly recognized as a valuable resource in agriculture [26]. By recycling this residue back into arable land, essential nutrients are returned to the soil, thus promoting the closure of nutrient and energy cycles. In detail, the application of biogas residue as a fertilization agent on arable land ensures that crops receive the majority of the essential nutrients required for growth [27]; in other words, soil fertility is conserved [28] and the soil structure and humus balance is improved, thus promoting closure of the natural nutrient and energy cycles. However, to ensure maximum recovery value, the application of biogas residue should have a meaningful purpose and optimal benefits [29]. Using biogas residue as a functional manure to inhibit pathogens offers a new approach.

Cotton (*Gossypium hirsutum* L.), as an important cash crop, is of great economic importance in many developing and some developed countries. *Verticillium* wilt, caused by the soil-borne fungal pathogen *V. dahliae*, results in dramatic losses in cotton yields in China. This destructive crop disease is hard to control because microsclerotia-fungal static structures may survive in the soil for more than 10 years [30]. Various cultivars differ in their susceptibility to *V. dahliae* [31]. Previous studies have shown that specific rhizosphere and endosphere microbes can contribute to resistance against *Verticillium* wilt in cotton. Beneficial bacteria, such as *Bacillales*, *Pseudomonadales*, *Rhizobiales*, and *Trichoderma*, have been associated with higher resistance and reduced disease development [32]. The tolerance of *Verticillium* wilt has been reported to be closely associated with well-known beneficial bacteria, including *Bacillus* [33], *Lysobacter* [34], *Streptomyces* [35], *Rhizobiales* [34], and *Pseudomonas* [36]. Therefore, incorporating biocontrol bacteria into crop fertilizers to control *Verticillium* wilt is a novel idea worth exploring.

A variety of microorganisms are present in kitchen fermentation residue. The residue used in this study additionally contained organic, inorganic, and mineral substances. Based on the source and possible pretreatments applied, the fermentation residue may contain microbial strains with special functions. In this study, strain *P. aeruginosa* ZL6 was obtained from kitchen fermentation residue and demonstrated strong antagonistic abilities against *V. dahliae*. The strain showed high identity to *P. aeruginosa*, a versatile bacterium known for its adaptability and colonization in various environments. The most closely related strain was *P. aeruginosa* JCM 5962^T^.

*Pseudomonas aeruginosa*, a species of g-proteobacteria in the family Pseudomonadaceae, is an opportunistic pathogen. It is a highly adaptable bacterium that can colonize various environmental niches, including soil and marine habitats, plants, animals, and humans [37]. Due to its genome’s large size and plasticity, *P. aeruginosa* is able to adapt to many situations and survive both the host immune response and antibiotic challenges [38]. One *P. aeruginosa* (CQ-4) was reported as potential biological control agents against the *Botrytis cinerea* in tomato and the strain has a clear promotion effect on tomato seed germination and seedling growth [39]. It is evident that *Pseudomonas* strains have been extensively studied for their biocontrol potential, including the suppression of fungal pathogens and promotion of plant growth. However, the specific biocontrol mechanisms of *Pseudomonas* against *V. dahliae* have not been widely reported. When studying its antibacterial mechanism, we observed that when *V. dahliae* and strain *P. aeruginosa* ZL6 were co-cultured on a PDA medium, the hyphae of *V. dahliae* were bent or even ablated (Fig. 1). This phenomenon piqued our interest. Upon reviewing the data, we found that the metabolites of *Pseudomonas* can affect the growth of *V. dahliae* [40]. Through further experimental research, we discovered that the metalloprotease of *P. aeruginosa* ZL6 has an antifungal rate on *V. dahliae*. It has been reported that *P. aeruginosa* produces an extracellular elastase that contributes to its pathogenicity [41]. The enzyme is a metalloprotease with a broad substrate specificity that encompasses biologically important host molecules such as elastin, collagen, transferrin, immunoglobins, and some complement components. The strain *P. aeruginosa* ZL6 produces the maximal metalloprotease Nml during the late-logarithmic and stationary growth phases. The metalloproteinase Nml was found to show strong inhibitory activity against the pathogenic fungi *V. dahliae* of cotton *Verticillium* wilt. As the key virulence factor of strain *P. aeruginosa* ZL6 against *V. dahliae*, the enzyme Nml had high homology with reported LasB elastase, which has been reported as a broad-spectrum exoprotease [42]. Recently, Everett *et al*. reported a LasB elastase inhibitors as potential drug targets and valuable tools for studying the proinflammatory impact of LasB in *P. aeruginosa* infections. Most importantly, they show clear potential for the clinical development of a novel therapy for life-threatening respiratory infections caused by this opportunistic pathogen [42].

The study also observed changes in the microbial community during the fermentation process after inoculating strain *P. aeruginosa* ZL6 derived from biogas residue. After inoculating the strain *P. aeruginosa* ZL6 derived from biogas residue and fermenting, the quantities and varieties of the microbes changed notably, as indicated by the metagenomics analysis. It was found that the diversity of the fermented biogas residue strains decreased, but the quantity of *Pseudomonas* rapidly increased. More interestingly, the quantity of *Bacillus* also increased significantly, indicating that the strain *P. aeruginosa* ZL6 may promote the proliferation of *Bacillus* during the fermentation process via interactions between bacterial strains. It has been reported that *P. aeruginosa* has extremely strong survival ability and can survive in exceedingly harsh environments, including in the presence of antibiotics, which could contribute to its influence on the microbial community. Moreover, *Bacillus* members have been reported as the preferred ideal biocontrol agents due to their capacity to form spores, enhance stress resistance in the soil, and their good inhibitory effects on various pathogenic bacteria. *Bacillus* spp., in addition to possessing a multitude of effect mechanisms of cooperation that can act synergistically against phytopathogens [43], have the capacity to form endospores to ensure their long-term maintenance and survival in different environments. The volatile organic compounds (VOCs) of the *Bacillus* members exhibit potential antagonistic behavior against several phytopathogens, including the pathogenic fungi *V. dahliae* [18].

The findings highlight the potential use of strain *P. aeruginosa* ZL6 from fermented biogas residue as a functional manure to inhibit *Verticillium* wilt. However, the application of biogas residue as a crop fertilizer and soil conditioner requires careful consideration and monitoring of soil properties, microbial activity, and plant growth [29, 44]. Therefore, the application of the residue to cropland not only requires rigorous post-treatment but also necessitates the monitoring of various factors including soil enzymatic activities and soil microbiology changes. Further research is needed to confirm the value and effectiveness of biogas residue as a crop fertilizer, emphasizing the importance of comprehensive studies in this field.

Overall, this study contributes to our understanding of the potential benefits and applications of biogas residue in agriculture, particularly in disease control and soil fertility improvement.

## Supplemental Materials

Supplementary data for this paper are available on-line only at http://jmb.or.kr.



## Figures and Tables

**Table 1:** Primers used in this study.

Primer name	Sequence(5’→3’)
CNm-F	GGATCTTCCAGAGATATGAAGAAGGTTTCTACGCTTGACC
CNm-R	CTGCCGTTCGACGATTTACAACGCGCTCGGGCA
TNm-F	GCCATGGCTGATATCGGATCCGCGGCCCAAGCGACATAA
TNm-R	CTCGAGTGCGGCCGCAAGCTTCTACCCGAAGGACTGATACGGC
KNm-F	AGGGAACAAAAGCTGGAGCTCACCCGCTACGAGCAATTCC
KNm-R	TTCCTGCAGCCCGGGGGATCCCTTGCTGTCGTTGGTGCTGC

**Table 2:** Results on the concentration, enzyme, and antifungal activities of the recombinant protein rm-Nml.

Protein Concentration (μg/ml)(SD)	Enzyme activity (PU) (×10^-3^) (SD)	Inhibition rate (%) (SD)
0.96 ± 0 .21^c^	4.23 ± 0.33^c^	30.10 ± 3.23^d^
1.64 ± 0.33^b^	5.33 ± 0.46^c^	48.87 ± 3.66^bc^
3.33 ± 0.35^a^	6.08 ± 0.49^bc^	40.22 ± 3.90^c^
4.75 ± 0.49^a^	7.57 ± 0.57^b^	53.52 ± 4.10^ab^
6.18 ± 0.65^a^	12.11 ± 1.16^a^	68.26 ± 4.69^a^

^a,b,c,d^Within a row means without a common superscript differ (*p* < 0.05).
