# Supplementary material for: Inhibition of Verticillium Wilt in Cotton through the Application of Pseudomonas aeruginosa ZL6 Derived from Fermentation Residue of Kitchen Waste
Source: J Microbiol Biotechnol. 2024 Apr 5;34(5):1040–50. doi: 10.4014/jmb.2401.01022 (PMC11180921; doi:10.4014/jmb.2401.01022)
Supplement: Supplementary file 1 [file jmb-34-5-1040-supple.pdf]

J. Microbiol. Biotechnol.
https://doi.org/10.4014/jmb.2401.01022

Supplementary Tables and Figures

Inhibition of Verticillium Wilt in Cotton Through the Application of *Pseudomonas aeruginosa* ZL6 Derived from Fermentation Residue of Kitchen Waste

Qiuhong Niu[1*], Shengwei Lei[1], Guo Zhang[2], Guohan Wu[1], Zhuo Tian[1], Keyan Chen[1], and Lin Zhang[1]

[1]College of Life Science and Agricultural Engineering, Nanyang Normal University, 1638 Wolong Road, Nanyang, Henan 473061, P. R. China

[2]College of Agriculture and Engineering, Nanyang Vocational College of Agriculture, Nanyang, Henan 473000, P. R. China

[*]Corresponding author: Qiuhong Niu

Table S1. Isolation, identification, characteristics, and inhibition activities of bacteria from the fermentation residue of kitchen waste.

Strain	Accessible number	Closest species in 16S rRNA gene sequence database	Similarity (%)	Inhibition activity (%)	Maximum growth temperature	Nitrogen fixation activity	Phosphorus solubilization activity	Potassium solubilization activity
ZL1	AM072764	*Exiguobacterium mexicanum* 8N^T	99.28	50.35	45°C	-	-	-
ZL2	JALV01000036	*Stenotrophomonas maltophilia* MTCC 434^T	98.77	20.04	40°C	+	-	-
ZL3	ACGZ01000098	*Lactobacillus plantarum* subsp. *Plantarum* ATCC 14917^T	99.86	38.90	42°C	-	-	+
ZL4	EU557337	*Pseudomonas yamanorum* 8H1^T	99.93	62.48	42°C	-	-	-
ZL5	JMPQ01000005	*Serratia marcescens* ATCC 13880^T	98.19	55.83	45°C	+	+	+
ZL6	BAMA01000316	*Pseudomonas aeruginosa* JCM 5962^T	100	95.31	42°C	+	+	-

Notes: - means negative; + means positive

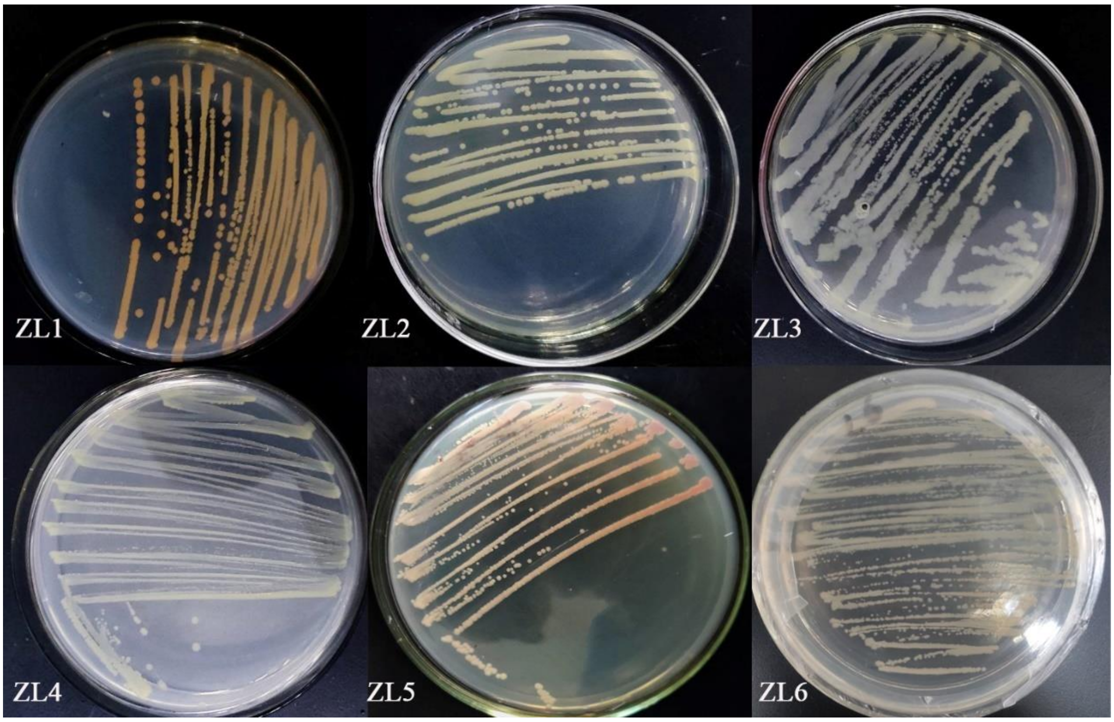

Fig. S1. Anti-fungal activity results of the six bacterial strains tested by plate inhibition experiments.

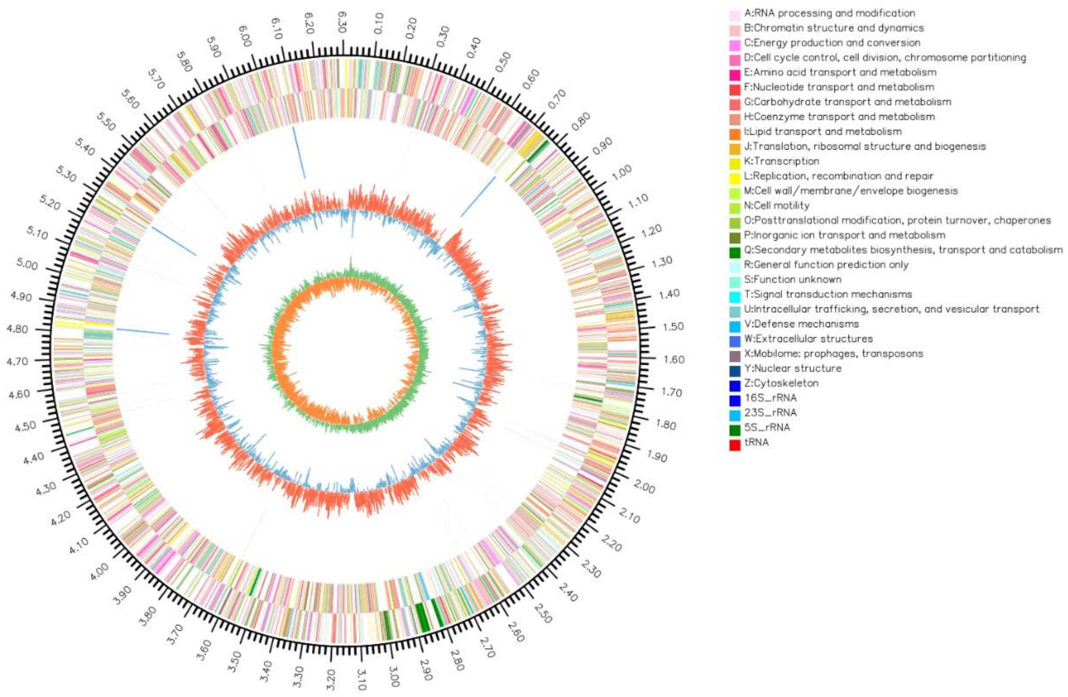

Fig. S2. Overview of the ZL6 genome. The outer circle is the position coordinate of the genome sequence, from outside to inside, which represents the annotation result of the coding gene and gene function, respectively.

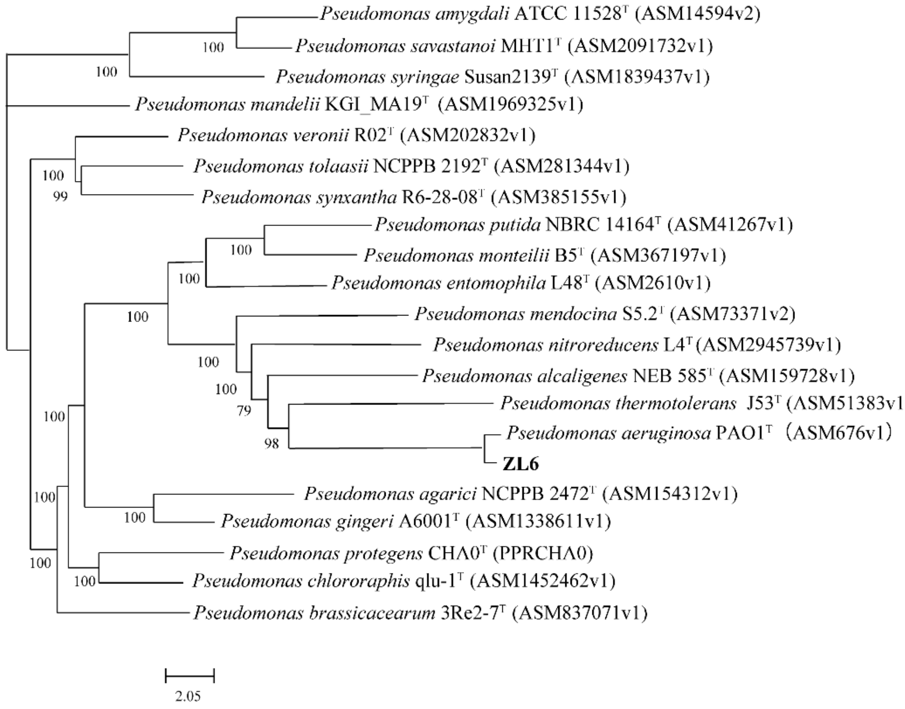

Fig. S3. Phylogenomic tree based on the core genomes of strains ZL6 and strains of related species of the genus *Pseudomonas*.

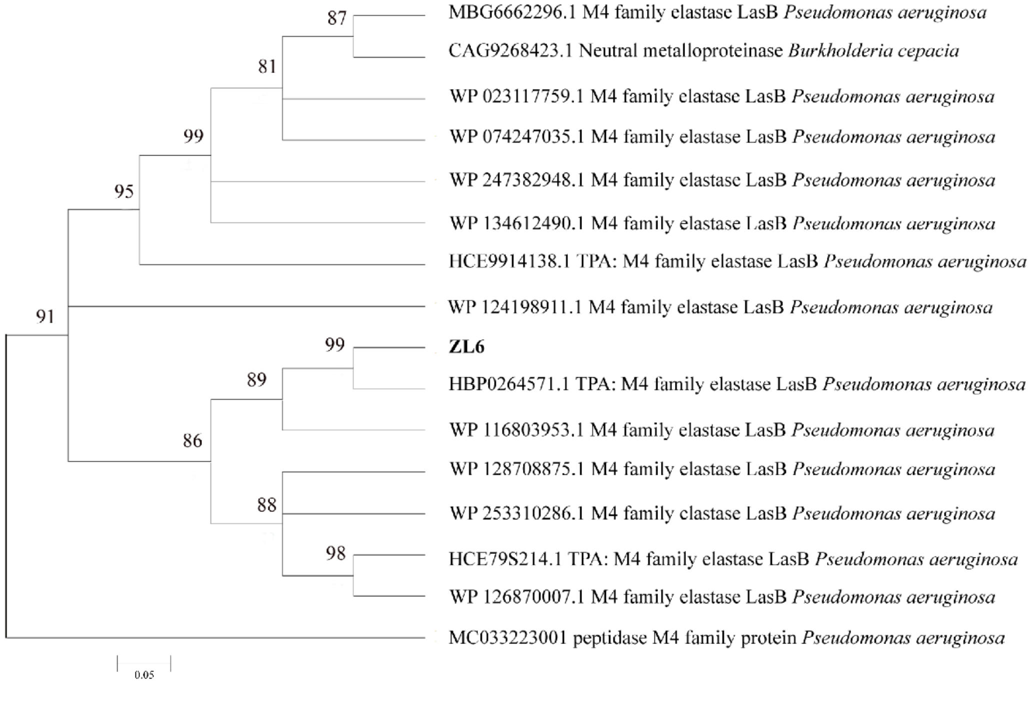

Fig. S4. Phylogenomic tree based on the amino acid sequences of the enzyme Nml of strain *P. aeruginosa* ZL6 and those from other related species.
